# CT abnormalities in late-onset schizophrenia and schizoaffective disorder correlate with number of psychotic episodes and cognitive dysfunction

**DOI:** 10.1192/j.eurpsy.2021.2133

**Published:** 2021-08-13

**Authors:** M. Savina, O. Bozhko, N. Savvateeva, V. Sheshenin, V. Pochueva, N. Cherkasov

**Affiliations:** Department Of Gerontological Psychiatry, FSBSI Mental Health Research Center, Moscow, Russian Federation

**Keywords:** late-onset psychosis schizophrenia neuroimaging

## Abstract

**Introduction:**

Late-onset psychosis is associated with development of dementia. Search for neuroimaging signs in these patients is important.

**Objectives:**

This study aimed to assess CT abnormalities and their clinical correlations in late-onset psychosis.

**Methods:**

Patients with DSM-V diagnosis of late-onset schizophrenia (LOS, n= 43, age 65.2±9.4, 90% females) and schizoaffective disorder (LOSAP, n=9, age 64.9±5.8, 30% females) underwent CT and cognitive examination before discharge. Atrophy and ventricles enlargement were ranged from 0 (abs.) to 3 (sev.); vascular pathology - from 0 (abs.) to 2 (mult.). Patients were compared with 16 controls (age 58.1±10.8, 50% females). Nonparametric statistic was used.

**Results:**

Patients had more severe frontal (χ^2^ 19.7, р=0.003), temporal (χ^2^ 10.7, р=0.097), parietal (χ^2^ 21.7, р=0.001), cerebellar (χ^2^ 14.8, р=0.005) atrophy and ventricles enlargement (χ^2^ 15.6 р=0.016). 29 % of LOS and 44% of LOSAP patients had leukoaraiosis. All findings correlated with age. In patients ventricular enlargement correlated with number of psychotic episodes (r=0.338, р=0.014), lower MMSE (r=-0.314, p=0.045), immediate (r=-0.508, р=0.002) and delayed (r=-0.404, р=0.016) verbal recall. Temporal atrophy correlated with number of episodes (r=0.439, р=0.001), lower MMSE (r=-0.327, p=0.037) and immediate verbal recall (r=-0.339, p=0.046); cerebellum atrophy - with lower MMSE (r=-0.338, р=0.036) and FAB (r=-0.407, р=0.01); leukoaraiosis - with number of episodes (r=0.503, р=0.001), prolonged hospital stay (r=0.345, p=0.024); vascular pathology – with number of episodes (r=0.336, р=0.015), lower visual recall (r=-0.399, р=0.019), performance time in TMT-B (r=0.404, р=0.024).

**Conclusions:**

Correlations between CT pathology, cognitive dysfunction and number of psychotic episodes may reflect progression of brain pathology due to psychosis.

**Disclosure:**

No significant relationships.

